# CENP-A Subnuclear Localization Pattern as Marker Predicting Curability by Chemoradiation Therapy for Locally Advanced Head and Neck Cancer Patients

**DOI:** 10.3390/cancers13163928

**Published:** 2021-08-04

**Authors:** Pierre Verrelle, Didier Meseure, Frédérique Berger, Audrey Forest, Renaud Leclère, André Nicolas, Emilie Fortas, Xavier Sastre-Garau, Marick Lae, Sabah Boudjemaa, Rodrigue Mbagui, Valentin Calugaru, Dalila Labiod, Leanne De Koning, Geneviève Almouzni, Jean-Pierre Quivy

**Affiliations:** 1 Institut Curie, PSL Research University, CNRS, Sorbonne Université, Nuclear Dynamics Unit, Equipe Labellisée Ligue Contre le Cancer, 26 rue d’Ulm, 75005 Paris, France; Audrey.Forest@curie.fr; 2 University of Clermont Auvergne, UFR Médecine, 63001 Clermont-Ferrand, France; 3 CNRS UMR 9187, INSERM U1196, Institut Curie, PSL Research University and Paris-Saclay University, 91405 Orsay, France; 4 Radiation Oncology Department, Institut Curie, 75005 Paris, France; rodriguembagui@gmail.com (R.M.); valentin.calugaru@curie.fr (V.C.); 5 Platform of Experimental Pathology PATHEX, Institut Curie, 75005 Paris, France; didier.meseure@curie.fr (D.M.); renaud.leclere@curie.fr (R.L.); andre.nicolas@curie.fr (A.N.); e.fortas@s-inter.com (E.F.); 6 Department of Diagnostic and Theranostic Medicine, Institut Curie, 75005 Paris, France; 7 Institut Curie, PSL Research University, Biometry Unit, 75005 Paris, France; frederique.berger@curie.fr; 8 Department of Pathology, Intercommunal Hospital Center of Creteil, 94000 Créteil, France; xavier.sastre@chicreteil.fr; 9 Department of Pathology, Centre Henri Becquerel, INSERM U1245, UNIROUEN, University of Normandie, 76031 Rouen, France; marick.lae@chb.unicancer.fr; 10 Department of Pathology, Hôpital Armand Trousseau, 75012 Paris, France; sabah.boudjemaa@aphp.fr; 11 Translational Research Department, Experimental Radiotherapy Platform, Institut Curie, PSL Research University, University Paris Saclay, 91400 Orsay, France; dalila.labiod@curie.fr; 12 Department of Translational Research, Institut Curie, PSL Research University, 75005 Paris, France; leanne.de-koning@curie.fr

**Keywords:** biomarker, head and neck cancer, chemoradiation therapy, histological imaging, chromatin and nuclear organization

## Abstract

**Simple Summary:**

For clinicians, rapid diagnosis of early neoplastic lesions and prediction of treatment response are two key aspects to guide their choice of treatment. Current histological markers are based on proliferation, differentiation states or specific cell function, but do not take full advantage of tumor characteristics. We show that the subnuclear distribution of CENP-A, the centromeric histone variant, provides, for both aspects, information distinct from and independent of commonly used markers. Our study reveals that in locally advanced head and neck squamous cell cancer patients, the subnuclear distribution of CENP-A at the time of diagnosis is an independent predictive marker of local disease control and curability by concurrent chemoradiation therapy. We provide evidence for the clinical applicability of this CENP-A labeling as a cost-effective marker regardless of genetic alterations in the tumor, perfectly compatible with the clinical time constraints in the course of therapy.

**Abstract:**

Effective biomarkers predictive of the response to treatments are key for precision medicine. This study identifies the staining pattern of the centromeric histone 3 variant, CENP-A, as a predictive biomarker of locoregional disease curability by chemoradiation therapy. We compared by imaging the subnuclear distribution of CENP-A in normal and tumoral tissues, and in a retrospective study in biopsies of 62 locally advanced head and neck squamous cell carcinoma (HNSCC) patients treated by chemoradiation therapy. We looked for predictive factors of locoregional disease control and patient’s survival, including CENP-A patterns, Ki67, HPV status and anisokaryosis. In different normal tissues, we reproducibly found a CENP-A subnuclear pattern characterized by CENP-A clusters both localized at the nuclear periphery and regularly spaced. In corresponding tumors, both features are lost. In locally advanced HNSCC, a specific CENP-A pattern identified in pretreatment biopsies predicts definitive locoregional disease control after chemoradiation treatment in 96% (24/25) of patients (OR = 17.6 CI 95% [2.6; 362.8], *p* = 0.002), independently of anisokaryosis, Ki67 labeling or HPV status. The characteristics of the subnuclear pattern of CENP-A in cell nuclei revealed by immunohistochemistry could provide an easy to use a reliable marker of disease curability by chemoradiation therapy in locally advanced HNSCC patients.

## 1. Introduction 

In the current era of precision medicine for cancer treatment, the need for biomarkers has become pressing in order to better select those patients that will benefit from a given treatment. 

Locally advanced head and neck squamous cell carcinoma (HNSCC) is a disease characterized by a low incidence of distant metastasis, for which concurrent chemoradiation therapy (CCRT) plays a major role in locoregional control and, consequently, curability. The choice between surgery followed by concurrent CCRT or exclusive CCRT remains a major and frequent issue. As major surgery can be mutilating, suboptimal and affects quality of life, CCRT is often selected as the first-line treatment with the risk of failing in controlling the disease. Active work is thus ongoing to search for markers predictive of locoregional disease curability by CCRT in order to guide this therapeutic choice [1,2,3]. HPV status detection is routinely used for HNSCC prognosis and tumor type classification [3,4]. Interestingly expression of the HPV-associated p16INK4A has been reported to be linked with response to radiotherapy in HNSCC [5,6]. Other markers of radioresistance have been reported in HNSCC [7,8] including disruptive P53 mutations as a potential candidate predicting low sensitivity to cisplatin-based chemotherapy and poorer survival [9]. Recent mutational signatures such as tobacco smoking identified in lung and head and neck tumors [10] cannot serve as predictive marker of response to therapy yet. Therefore, there is currently no validated marker routinely used in clinics to discriminate those patients that are curable by CCRT from those that are not. 

Chromatin and nuclear organization have long been linked to genome function and cancer [11,12,13,14,15,16]. Thus, the recent capacity to target chromatin and its regulators has broadened possibilities of conventional treatments (chemotherapy, radiotherapy) [17,18,19]. Here, we aimed at characterizing a novel biomarker of clinical interest for both diagnostic and prognostic purposes by probing key features in chromatin and nuclear organization. We focused on the centromeric histone H3 variant CENP-A [20,21]. Indeed, CENP-A marks centromeres which are key chromatin regions to enable proper chromosome segregation at each cell division [22,23]. Furthermore, outside mitosis, centromeres contribute to genome architecture as robust nuclear domains [11,24]. They display distinct features in nuclei during the acquisition of specific cell fate [25,26,27]. Thus, localizing centromeres provides a means to monitor major re-organization in nuclear architecture. Since CENP-A is a universal marker of the centromere, probing CENP-A localization represents an advantageous proxy to follow changes in nuclear organization. Importantly, CENP-A expression has been linked to cancer. In several human cancers, including breast [28,29], colorectal [30], liver [31], lung [32], ovarian [33] and osteosarcoma [34], CENP-A overexpression and/or increased amounts have been reported. Interestingly, CENP-A expression levels reflect various responses to radiation at a cellular level [35,36] that are potentiated by the p53 status of the cells [37]. In addition, in a large series of breast, lung, ovarian and gastric cancer patients, a score quantifying the overexpression of 14 centromere and kinetochore genes, comprising CENP-A, has been associated with poor patient survival and higher risk of disease progression, but is also predictive for improved patient response to adjuvant chemo- and radiotherapy [38]. These studies underlined a significant role of CENP-A expression levels in tumors and response to therapy considering global amounts of CENP-A. However, the feature of the spatial distribution of CENP-A had not been considered in patient samples. 

We thus hypothesized that the subnuclear patterns corresponding to CENP-A localization in the nucleus could provide clinically relevant information for both diagnostic and prognostic purposes. 

We report the first identification of distinct staining patterns for CENP-A in the nucleus of any healthy and tumoral human tissue, indicating that CENP-A subnuclear localization is a potential marker of neoplastic transformation. We performed a retrospective study by visualizing CENP-A subnuclear patterns in pretreatment biopsies from a cohort of 62 HNSCC patients treated by concurrent chemoradiation therapy (CCRT). We found that a distinct CENP-A pattern correlated with tumor control at >2 years and patient survival with a high significance and that it is a predictive marker of curability of HNSCC patients by CCRT. Taken together, our data indicate that the subnuclear pattern of CENP-A is a valuable diagnostic, predictive and prognostic marker to refine tumor characterization and treatment choice for the benefit of patients.

## 2. Results

### 2.1. CENP-A Clusters in Foci at the Nuclear Periphery in Normal Human Tissue Fixed with AFA

**Figure 1:**
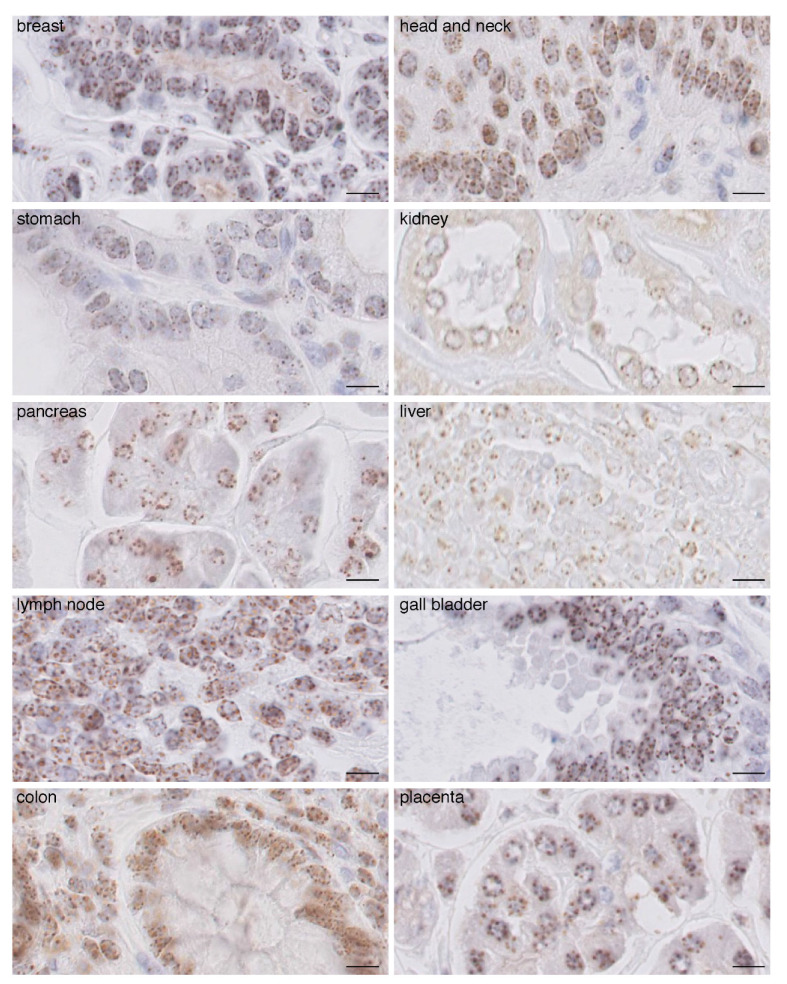
CENP-A staining by IHC in normal human tissues. Images of CENP-A staining by IHC in the indicated human tissues. Scale bar is 10 µm.

To perform immunohistochemistry (IHC) staining to visualize CENP-A in cell nuclei in paraffin-embedded human tissues, we first assessed tissue fixation in order to ensure proper access of the antibodies in order to recognize the CENP-A epitope in the nucleus. While standard formol fixation procedure did not allow us to obtain a consistent and reliable signal using two distinct anti-CENP-A antibodies (Supplementary Figure S1A), fixation with alcohol–formalin–acetic acid (AFA) yielded a robust and optimal CENP-A signal with identical profiles for both antibodies (Supplementary Figure S1A). Then, using normal tissues of different origin fixed in AFA, we found that although CENP-A staining varies among tissues in terms of signal intensity, in all tested tissues, CENP-A localized at the nuclear periphery as individual equidistant round foci of similar size, estimated in the range of 0.6 microns (Figure 1). In fewer cases, we also detected CENP-A foci at the periphery of the nucleolus. This localization was remarkably conserved and did not correlate with signal intensity for CENP-A. We detected, per section, an average of 5.5 equidistant round foci per nucleus in every tissue (range is four to eight foci per section). Assuming an average nuclear diameter of 5 to 10 microns and 3 micron thick sections, the number of foci per nucleus can be estimated in the range of 9–18 foci/nucleus. The normal human genome is composed of 46 chromosomes (22 pairs of autosomes and two sexual chromosomes) per nucleus in diploid cells. Therefore, we would expect 46 CENP-A foci if all centromeres were separated. Our data thus indicate that, in interphasic cells, several centromeric CENP-A-rich regions from different chromosomes cluster together to form foci, with an estimation of two to five chromosomes on average per foci. In order to eliminate the possibility of artefactual CENP-A patterns due to paraffin embedding and IHC procedures, we also performed cryo-sections and carried out CENP-A staining and analysis by immunofluorescence on fresh frozen cryo-preserved healthy breast tissue. Similar to IHC, we found that in the nucleus, CENP-A foci localization is restricted at the nuclear periphery in a single focal plane (Supplementary Figure S2A) and in every consecutive focal plane from confocal acquisition (Supplementary Figure S2B). From the 3D confocal acquisition, we found an average of 10 foci per nucleus (Supplementary Figures S3A and S4A, #a non-tumoral), in line with our findings above when detecting CENP-A by IHC. Thus, taken together, IHC and immunofluorescence detection of CENP-A indicate that in all of the healthy tissue we analyzed, CENP-A localization follows a well-defined and conserved pattern characterized by nine to 18 individual equidistant foci that are homogenous in size and round, all positioned at the nuclear periphery within each nucleus (see ‘normal’ scheme in figure below).

### 2.2. In Carcinomas CENP-A Clustering and Localization at the Nuclear Periphery Are Altered

**Figure 2:**
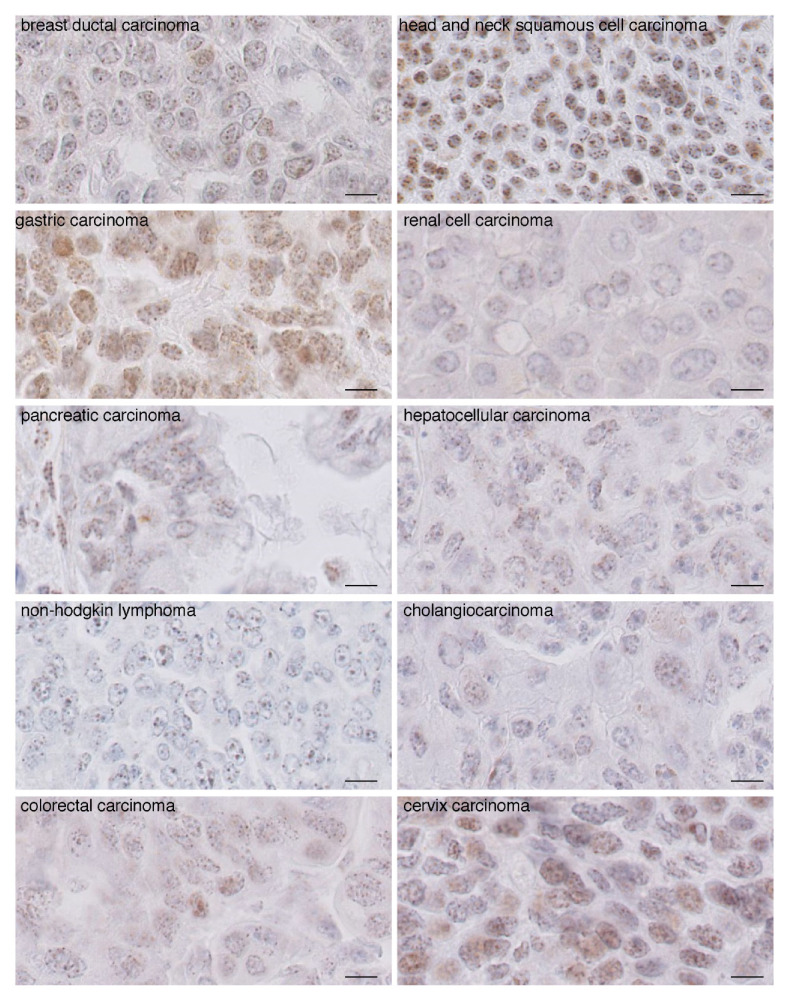
CENP-A staining by IHC in carcinomas reveals a pattern distinct from that of non-tumoral tissue. Images of CENP-A staining by IHC in non-Hodgkin lymphoma and carcinomas as indicated. Scale bar is 10 µm.

We next performed CENP-A IHC staining on carcinomas of the same tissue origin as the normal tissue above (Figure 2). We found that the intensity of CENP-A staining increased in most, but not all, tumor tissues compared to non-tumoral tissues (compare Figure 1 and Figure 2), in agreement with reported overexpression of CENP-A in tumors. Most strikingly, we found that CENP-A foci do not localize strictly at the nuclear periphery but, rather, are detected inside the nucleus, although a few foci remain at or in the vicinity of the nuclear periphery. Second, both the round shape and equidistant localization of the foci are affected, leading to a decreased homogeneity within and among the different nuclei. Third, the number of foci per nucleus increases concomitantly with a reduction in their size (Figure 2). We also produced cryo-sections and carried out CENP-A staining and analysis by immunofluorescence on fresh frozen breast carcinomas (Supplementary Figures S3 and S5). Using confocal microscopy to monitor CENP-A localization across the entire nucleus, we also found that CENP-A foci localization is not restricted to the nuclear periphery as in normal tissue (Supplementary Figure S5). Compared to healthy breast tissue, we detected a decreased size of the individual CENP-A foci, an increased number of CENP-A foci per nucleus (Supplementary Figures S3 and S4A) and a broader range of the number of CENP-A foci per nucleus (Supplementary Figure S4B). Taken together, both IHC and immunofluorescence data indicate that in carcinomas, both CENP-A clustering and localization at the nuclear periphery are altered, ultimately leading to a diffusive intranuclear staining pattern, that can be straightforwardly distinguished from that of healthy tissue (Figure 2). Having established that changes in CENP-A nuclear localization pattern reflect neoplastic states, we next monitored how CENP-A localization patterns change with tumorigenesis onset and progression.

### 2.3. Change in CENP-A Nuclear Localization Pattern as a Marker of Malignancy

**Figure 3:**
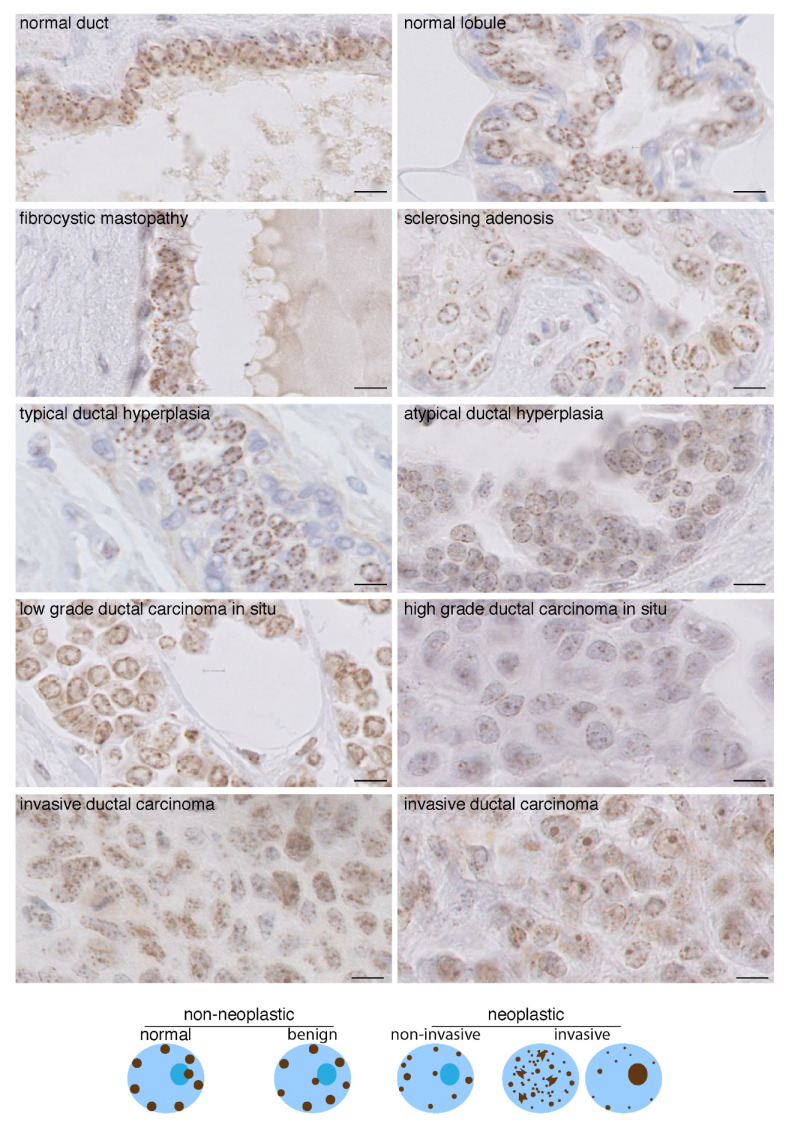
CENP-A localization patterns in breast lesions. Top: CENP-A staining by IHC in breast tissues as indicated. Invasive ductal carcinoma displaying CENP-A foci localized only inside the nuclear space (**left**) and a few remaining at the nuclear periphery (**right**) are shown. Scale bar is 10 µm. Bottom: scheme depicting the patterns.

We used breast tissues and compared CENP-A patterns in samples corresponding to benign non-neoplastic breast lesions (dystrophic and simple hyperplastic: fibrokystic disease, sclerosing adenosis and typical hyperplasia) and neoplastic lesions of increasing malignancy ranging from atypical hyperplasia (AH) to ductal carcinoma in situ (DCIS) and invasive ductal carcinoma (IDC) (Figure 3). We found that in benign lesions, CENP-A localizes in the nucleus of all epithelial cells as four to eight equidistant foci, of the same size (0.6 micron) and with a round shape, most often at the nuclear periphery and sometimes at the nucleolus periphery. This localization pattern is similar to that of normal tissue (Figure 1 and Figure 3). In contrast, this distinct pattern is lost in AH, DCIS and IDC. In AH and DCIS, we observed that, in the majority of atypical epithelial cells, CENP-A foci had a reduced size and sometimes were even not detected, not restricted only at the nuclear periphery and not equidistant (Figure 3). These changes became more prominent in IDC, with CENP-A foci most frequently absent from the nuclear periphery but localized inside the nuclear space. Furthermore, the number of these CENP-A foci localized inside the nuclear space is usually larger, whereas their size decreases and their shape within a single nucleus becomes heterogeneous (Figure 3), indicative of a loss of clustering, as observed above in carcinomas from different tissues (Figure 2). Finally, we frequently identified in the IDC samples a combined CENP-A signal at the nuclear periphery, inside the nuclear space, and perinucleolar localization, leading to heterogeneous aspects among the nuclei (Figure 3 and schemes below). Interestingly, we found that in contrast to low-grade DCIS, the high grade displayed a pattern similar to or resembling that of IDC. 

Taken together, these data indicate that various parameters change in CENP-A localization in the nucleus of cells during oncogenic transformation/progression: (i) decreased clustering of CENP-A foci (decreased size and increase in foci number) associated with progressive decrease of CENP-A localization at the nuclear periphery discriminates benign dystrophic and hyperplastic breast lesions from in situ and invasive neoplastic breast lesions (AH, DCIS and IDC); (ii) loss of CENP-A localization at the nuclear periphery and heterogeneity in terms of number, size and shape of CENP-A foci localized inside the nuclear volume within and among nuclei discriminates invasive neoplastic (IDC) from non-invasive breast lesions (AH, DCIS) (Figure 3 schemes).

Given that the amount of CENP-A has been linked to radiation response in cells [36,37] and radiation therapy in patients [38], we investigated whether parameters of the CENP-A localization pattern could also be linked to radiation response.

### 2.4. CENP-A Nuclear Localization in Radioresistant and Radiosensitive HNSSC-Derived Cell Line Xenografts

We used the two human cancer cell lines, SCC61 and SQ20B, derived from HNSCC that display radiosensitive (SCC61) and radioresistant (SQ20B) behaviors [39]. First, in order to mimic in vivo tumor growth and cellular three-dimensional constraints that can modulate radioresistance [40], we generated subcutaneous SCC61 and SQ20B xenografts in nude mice and verified that following a 5 × 4 Gy fractionation, SCC61-derived tumors were sensitive to radiation, in contrast to the SQ20B (Supplementary Figure S6A). Next, we monitored the CENP-A localization pattern in SCC61 and SQ20B tumors prior to irradiation by immunofluorescence staining. We found for both cell lines a CENP-A pattern that displayed characteristics of neoplastic lesions, including decreased CENP-A clustering and the absence of systematic localization at the nuclear periphery (Supplementary Figure S6B). Remarkably, we found that the radiosensitive cell line SCC61 displayed a similar intensity and pattern within and amongst every nucleus. In contrast, the CENP-A pattern in the radioresistant SQ20B cell line appeared very heterogeneous (Supplementary Figure S6B), suggesting that distinct CENP-A patterns might be associated with radiosensitive or radioresistant properties. We next investigated, in patient samples, whether specific CENP-A patterns in HNSCC patients were also associated with a particular response to CCRT. 

### 2.5. CENP-A Nuclear Localization Pattern Is Associated with Response to CCRT

We selected 62 consecutive HNSCC patients treated at Institut Curie by CCRT without surgery as a first-line treatment, for which clinicopathological data and pretreatment biopsies fixed in AFA were available (Supplementary Table S1). TNM and stages of different specific localization sites are detailed in Supplementary Table S2. Among these 62 patients, 38 (61.3%) patients showed a locoregional control persisting at 2 years, while 24 (38.7%) patients suffered from in-field locoregional progression within 2 years (Supplementary Table S1). In this cohort, gender, age of diagnosis, T (TNM), N (TNM), stage, and tumor site did not impact local disease control at 2 years. Variability in treatment regimen, such as the administration of induction chemotherapy, temporary (>5 days) CCRT interruption (7 patients, 11.3%), total delivered radiation dose or the protocol of concurrent chemotherapy did not impact the disease control either (Supplementary Table S1). Only one metastatic relapse was observed for a patient in which a local control was achieved (2.6%) compared to 11 (47.8%) for patients without locoregional control (*p* < 0.001) (Supplementary Table S1). 

**Figure 4:**
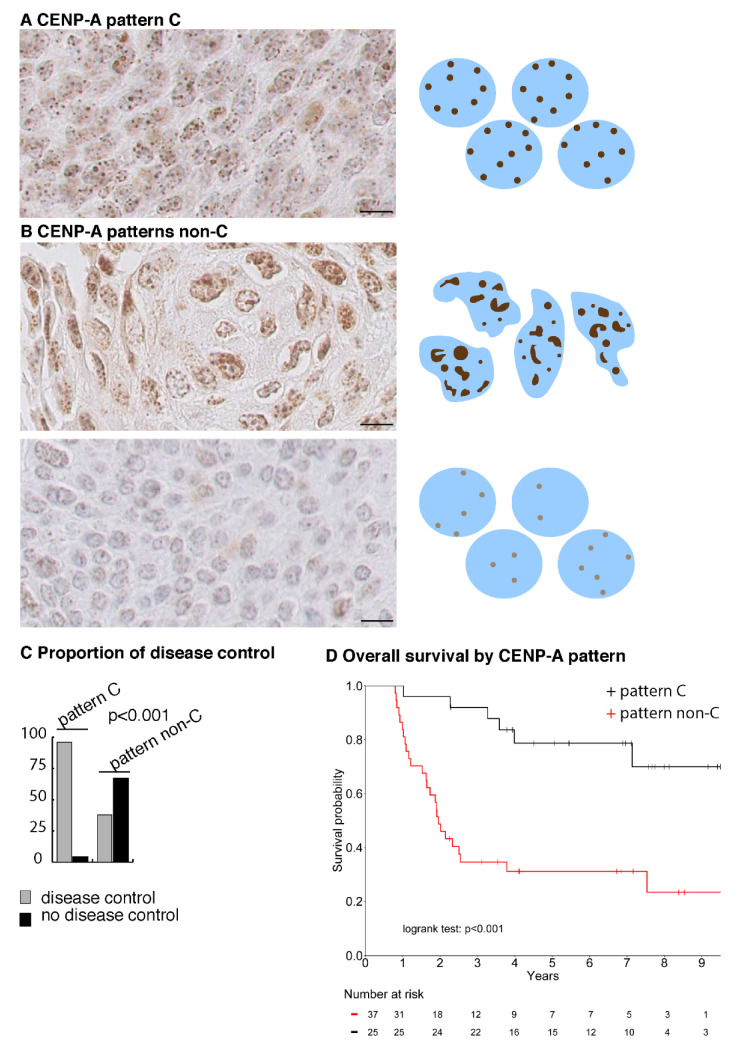
CENP-A nuclear localization pattern is a marker of disease control by CCRT in HNSCC patients. (**A**) CENP-A IHC images of HNSCC biopsies corresponding to CENP-A pattern-C. Scale bar is 10 µm. A scheme depicting the pattern is shown on the right (**B**) as in (**A**), but for CENP-A pattern non-C. (**C**) Graph showing proportion (in %) of disease control in biopsies from patients displaying CENP-A pattern-C or non-C. (**D**) Kaplan–Meier survival curve of patients treated by CCRT with CENP-A pattern-C (black) and CENP-A pattern non-C (red).

We performed CENP-A IHC staining on the biopsies and identified a clear and distinct discriminative pattern (Figure 4A). This pattern is first characterized by its homogeneity that can be appreciated at all levels: the number, size, shape, localization and intensity of CENP-A foci appear similar for every nucleus of the section (Figure 4A). Second, it combines predominant localization inside the nuclear space and few localizations at the nuclear periphery of CENP-A foci, with strong to medium CENP-A immunostaining intensity and mild anisokaryosis. We designated this pattern as pattern-C (Figure 4A). Conversely, patterns that do not belong to pattern-C, or pattern non-C, displayed heterogeneity within and amongst nuclei of the section in terms of nuclear localization, intensity, size, shape and number of CENP-A foci, with variable staining intensity and moderate to marked anisokaryosis (Figure 4B). Intriguingly, pattern-C is similar to that of the radiosensitive cell line SCC61-derived tumors, and pattern non-C is similar to that of the radioresistant SQ20B-derived tumors (compare Supplementary Figure S6B with Figure 4A,B). Following examination by three pathologists, blinded to the response of the patients to CCRT, we could split the 62 biopsies into two groups: displaying with CENP-A staining either pattern-C or pattern non-C. We found that, in patients displaying pattern-C, the disease is controlled in 96% (24/25) of cases, while pattern non-C was associated with locoregional progression in 62.2% (23/37) of patients (*p* < 0.001), in line with our observations with the SCC61 and SQ20B cell lines (Figure 4C and Table 1). Next, we assessed the correlations between patients’ response to CCRT, tumor characteristics, CENP-A nuclear localization pattern (C or non-C), amount of CENP-A (H-score), proliferation (Ki67), anisokaryosis and HPV status (p16). Ki67 is not correlated with response to CCRT. In contrast, as expected, 26 (68.4%) HPV-positive (HPV+) patients achieved control of their disease against only 12 (44.4%) HPV-negative (HPV−) patients (*p* = 0.02). Patients who achieved local control displayed mild anisokaryosis in 63.2% of cases while 83.4% of relapsing patients showed moderate to marked anisokaryosis (*p* < 0.001). Interestingly, the CENP-A pattern-C is associated with a significantly higher CENP-A H-score compared to pattern non-C (*p* = 0.003) and 80% of CENP-A pattern-C patients are HPV+ (*p* = 0.002) and display mild anisokaryosis (*p* < 0.001) (Table 1). It is important to note that patient’s age, gender, tumor stage, tumor site and Ki67 level do not differ according to the CENP-A staining pattern (Table 1). 

### 2.6. A CENP-A Nuclear Localization Pattern Is a Marker of Predictive Value for Curability of HNSCC Patients by CCRT

We next investigated the predictive value of those variables for local disease control, first using a univariate logistic regression model testing the probability of local disease control at two years. A positive HPV status (OR = 3.6, 95% CI [1.3; 11.0]), *p* = 0.02), a higher CENP-A H-score (OR = 3.1, 95% CI [1.5; 7.2], *p* = 0.005) and CENP-A pattern-C (OR = 39.4, 95% CI [7.1; 744.0], *p* = 0.001) are significantly associated with disease control, while moderate to marked anisokaryosis is associated with disease recurrence (moderate vs. mild: OR = 0.3, 95% CI [0.1; 1.1], marked vs. mild: OR = 0.03, 95% CI [0; 0.1], *p* < 0.001) (Table 2). In multivariate analysis, a low level of anisokaryosis (moderate vs. mild: OR = 0.7, 95%CI [0.1; 3.9], marked vs. mild: OR = 0.1, 95% CI [0.01; 0.7], *p* = 0.03) and the presence of CENP-A pattern-C (OR = 17.6, 95% CI [2.6; 362.8], *p* = 0.002)) remain independent predictive factors for local disease control at two years (Table 2). Among the controlled patients, 24/38 had CENP-A pattern-C, leading to sensitivity of CENP-A pattern-C of 63.2% (95% CI [46; 78.2]). Among the non-controlled patients, 23/24 did not have CENP-A pattern-C, corresponding to a specificity of 96% (95% CI [78.9; 99.9]) of CENP-A pattern non-C (Supplementary Table S1). Interestingly, within the subpopulation of 35 HPV+ patients, who intrinsically have a better disease control than HPV- patients (Supplementary Table S1), CENP-A nuclear localization pattern remains an independent predictive factor of local control at 2 years (OR = 9.2, 95% CI [1.02; 203.2], *p* = 0.048). 

In line with the predictive value of the CENP-A pattern-C for local disease control by CCRT, CENP-A pattern-C-positive patients demonstrate a significantly better overall survival at 5 years (79%; 95% CI [64%; 97%]) compared to CENP-A pattern non-C patients, (31%; 95% CI [19%; 51%]) (log rank test *p* < 0.001) (Figure 4D).

Taken together, our results in HNSCC show that CENP-A nuclear localization pattern-C is a new predictive marker of local disease control at two years following CCRT, independent of HPV status, and is highly prognostic for overall survival.

## 3. Discussion

We found that both CENP-A clustering as foci with a unique localization of these foci restricted at the nuclear periphery define a nuclear pattern conserved in every normal solid tissue that we analyzed. Given that similar observations are reported in human lymphocytes [41,42,43,44] this unique organization of CENP-A is likely to be a fundamental feature of chromatin and nuclear organization in every healthy cell, irrespective of their tissue of origin or differentiation pathway, and is in line with the hypothesis that centromeric regions are robust and stable chromosomal loci [11,24]. We found that this fundamental organization is also maintained in benign breast lesions, but lost in neoplastic states. This therefore positions the CENP-A nuclear localization pattern as a marker of potential interest in the context of neoplastic transformation. 

Most importantly, we also established in this retrospective study that a specific pattern of CENP-A nuclear localization, that we named CENP-A pattern-C, is a marker of predictive value for local disease control by CCRT in HNSCC patients.

The mechanisms involved in the clustering of centromeres and the control of nuclear organization are still largely unknown [24,45] and whether the predictive value of the pattern is driven by CENP-A-specific functions, or not, remains to be determined. Given links between p53 and CENP-A expression [37,46], how these CENP-A changes interplay with the p53 status will be attractive to explore. Furthermore, the predictive value of CENP-A pattern-C may also be independent of CENP-A-functions. Indeed, it is also possible that other parameters impacting chromatin and nuclear organization could impact CENP-A localization in the nucleus. In any event, CENP-A localization as a read out can serve as a marker highlighting cancer-related chromatin and nuclear disorders. In this respect, it is also interesting to note that CENP-A pattern-C reflects a less transformed and disorganized neoplastic state, possibly intrinsically more sensitive to CCRT. 

Importantly, HNSCC is curable by CCRT providing that the tumor is controlled locally, therefore positioning CENP-A pattern-C as a predictive marker of curability by CCRT, and, consequently, a prognostic marker. Our results show that CENP-A pattern-C is not correlated to Ki67 and confirm that proliferation is not significantly associated with response to CCRT ([8] and references therein). They also confirm that HPV-positive status is associated with a better prognosis for response to CCRT and patient survival [3,47]. This has led to the distinction of HNSCC according to HPV status in the last UICC TNM classification in 2017 [48]. However, despite this improved prognosis, some HPV-positive patients still relapse [49] and, consequently, HPV positivity cannot be used as a discriminatory criterion to identify HNSCC patients curable by CCRT. Most importantly, our results show that CENP-A pattern-C is independent of HPV+ status and has a higher probability to identify curable patients compared to HPV-positive status (Table 2). In addition, we found that CENP-A remains predictive for response to CCRT among HPV-positive patients.

We believe that IHC-based CENP-A labeling can easily be implemented in routine clinical practice, providing that samples are fixed in AFA. As a marker of chromatin and nuclear organization, it offers for the clinician a novel parameter distinct from those that are typically used, such as proliferation, differentiation status or specific cell type and function, which should help to refine diagnosis of neoplastic lesions. In HNSCC patients, prediction of curability by CCRT at the time of diagnosis represents a major advance that should contribute to choosing the most appropriate treatment between conservative CCRT or surgery-based protocols. Finally, due to the fundamental organization of CENP-A foci conserved in every tissue examined, CENP-A pattern could be considered for predicting response to CCRT in other clinical situations, such as anal carcinomas, cervix carcinomas and locally advanced non-small cell lung or esophagal carcinomas that are potentially curable by CCRT. Together, these cases represent >1M per year worldwide. Future studies with distinct cohorts should aim at validating our results and extending them to other cancers. For prediction of disease response to chemoradiation and patient outcome, automated detection and analysis of CENP-A patterns, together with deep learning-based approaches [50], will be key to facilitating the clinical implementation of this new biomarker.

## 4. Methods

Detailed procedures for detailed processing of tissue sections for immunohistochemistry staining; immunofluorescence staining and image acquisition and analysis; xenografts; and irradiation are provided as Supplementary Information.

### 4.1. Tissue Samples

Biopsies used in the study were fixed in alcohol, formaldehyde and acetic acid (AFA) at collection. We analyzed samples from 16 types of primary malignant tumors (*n* = 628) and 16 types of normal tissues (*n* = 249) collected from multi-center departments of pathology from 1978 to 2010. For breast tissues, we analyzed samples from (i) 10 normal breasts, (ii) 30 benign breast lesions, including sclerosing adenosis atypical (*n* = 10), fibrocystic disease (*n* = 10) and simple ductal hyperplasia (*n* = 10), (iii) 30 preinvasive breast lesions, including atypical columnar metaplasia (*n* = 10), atypical ductal hyperplasia (*n* = 10) and ductal carcinoma in situ (*n* = 10) and (iv) 150 invasive breast carcinomas (IBCs). 

### 4.2. Immunohistochemistry Imaging

We made 3 micrometer thick sections from paraffin-embedded tissue blocks, obtained at the time of the initial diagnosis (before treatment) and fixed in AFA. Detailed processing of tissue sections is provided as Supplementary Information. All immunohistochemistry stainings were processed using a Leica BOND RX research automated immunostaining device. Antibodies used were: anti-CENP-A, Cell Signaling #2186, 1/50, pH = 9; anti-Ki67, DAKO M7240 clone MIB-1, 1/200, pH = 9; anti-CENP-C Clinisciences #PD03 1/50, pH = 9; anti-H3K9me3 active motif #39765 1/50, pH = 9. For controlling CENP-A pattern specificity, we used anti-CENP-A, Enzo Life Sciences #ADI-KAM-CC006-E 1/50, pH = 9. For comparing CENP-A patterns between AFA and formol fixation, we used paraffin-embedded tissue blocks fixed in formaldehyde. We performed image acquisition using a Philips IMS Ultra-Fast Scanner 1.6 RA. Frequency of CENP-A and Ki67 staining expressed as % of positively detected nuclei was obtained by visual examination of 100 nuclei in 50 different fields. H-score (intensity x frequency) was calculated from frequency of CENP-A-positive cells and CENP-A signal intensity was quantified using three staining scoring categories (1: weak, 2: moderate and 3: strong). Anisokaryosis was assessed from counterstaining as 1: mild, 2: moderate and 3: marked. Images were scored by 3 experienced pathologists. 

### 4.3. HNSCC Patients

The main inclusion criteria in the study were (i) diagnosis of non-metastatic locally advanced HNSCC between 2007 and 2015, according to the UICC TNM classification 2011 [51], (ii) treatment with a curative and conservative intent by CCRT, preceded or not by induction chemotherapy and (iii) availability of pretreatment tumor samples collected at initial diagnosis and fixed in AFA. In total, 62 consecutive patients were included in the study. We used HPV status from initial diagnostics and determined Ki67 status from the original primary biopsies of the 62 HNSCC patients by immunohistochemistry staining. The median age at diagnosis was 62 years (range: 56–68), 52 patients (83.9%) were males. Tumors were mainly located in the oropharynx (67.7%), the other locations were larynx (17.7%), oral cavity (8.1%) and hypopharynx (6.5%). Tumors were classified as stage IVa or IVb in 64.5%, stage III in 29%, stage II in 4.8% and stage I in 1.6% of cases. Thirty-five patients (56.5%) had HPV-positive tumors. An induction chemotherapy was proposed for 24 patients (38.7%); 16 (66.7%) of them received TPF (docetaxel–cisplatin–fluorouracil), while the others received either a combination of taxol–carboplatin (6 patients, 25%) or taxol–carboplatin–evelorimus through a clinical trial (2 patients, 8.3%). All patients underwent radiotherapy, 60 (96.8%) at a dose of 70 Gy and 2 (3.2%) at a dose of 74.2 or 74.4 Gy. In 42 (67.7%) cases, the radiotherapy was combined with concurrent chemotherapy or targeted therapy (either cisplatin (54.87%) or cisplatin-5FU (2.4%) or carboplatin (2.4%) or cetuximab (40.5%)). At the time of data collection, the median follow-up was 7 years (range 2.3–9.6 yrs) and the median overall survival was 7.1 years (95% CI [2.5; Not reached]. Patient and disease characteristics and treatment are also detailed in Supplementary Tables S1 and S2.

### 4.4. Statistical Analysis

Due to the exploratory nature of the study, we could not make an upfront hypothesis regarding the number of cases needed to achieve the study’s objectives; we did not hierarchize objectives into primary or secondary, nor did we specify any power to be reached. Detailed procedures are described in Supplementary Material and Methods. HNSCC patient characteristics are presented as the mean with standard deviation (SD) when normally distributed, or as the median with range (minimum and maximum) in the case of skewed data. Categorical data are presented as numbers and proportions. Differences between continuous variables were assessed using a Student’s *t*-test or Mann–Whitney U test, depending on normality, whereas the chi-squared test or Fisher exact test were used for categorical values. Overall survival is defined as the time between the date of diagnosis and the date of death, patients alive were censured at their date of last news. Median of follow-up and survival curves were estimated using the Kaplan–Meier method and compared by a log-rank test. A logistic regression model was used with the local disease control at 2 years as an outcome variable and included baseline patient characteristics, HPV status, anisokaryosis, CENP-A H-score and CENP-A nuclear localization pattern (CENP-A pattern non-C or CENP-A pattern-C) as covariates. Factors with a significant *p*-value less than 10% in the univariate analysis were included in a multivariate stepwise top-down procedure using the Akaike information criterion (AIC) and the likelihood ratio test as criteria for variable selection. The corresponding odds ratios (OR) was calculated with the 95% confidence interval (95%CI). A *p*-value less than 0.05 is considered statistically significant. To evaluate the predictive value of CENP-A, we performed the analysis in line with the REMARK guidelines. All the analyses were performed using R software version 3.6.2 (R Core Team (2019) (R: A language and environment for statistical computing. R Foundation for Statistical Computing, Vienna, Austria. https://www.R-project.org/, accessed on 07/08/2020).

## 5. Conclusions

Our results indicate that the identification of distinct CENP-A subnuclear patterns represents a novel biomarker of interest relying on three-dimensional properties of chromatin in the nucleus, providing information distinct from other commonly used biomarkers in oncology. In the context of locally advanced HNSCC, a specific subnuclear pattern is predictive of curability by CCRT. Our findings could have important implications for the choice of therapeutic options, including conservative CCRT or surgery-based protocols, for locally advanced HNSCC patients.

## 6. Patent

Results described here are covered by a European Patent Application number EP21305439, filed on 6 April 2021. ‘Methods and Kits for Diagnosing Cancer and Predicting Response to Treatment Based on Cenp-A Labelling’.

## Figures and Tables

**Table 1:** Correlations between CENP-A pattern-C and non-C and patients and tumor characteristics.

Characteristic		Total (*N* = 62)	CENP-A Pattern		
			C (*N* = 25)	Non-C (*N* = 37)	Test
**Age (year) **					NS
	Median (IQR)	62 (56–68)	61 (56–64)	63 (56–69)	
**Ki67 (%) **					NS
	Median (IQR)	60 (35–75)	60 (35–75)	60 (30–75)	
**Gender N (%) **					NS
	F	10 (16.1%)	6 (24%)	4 (10.8%)	
	M	52 (83.9%)	19 (76%)	33 (89.2%)	
**T (TNM) N (%) **					0.06
	T1	4 (6.5%)	1 (4%)	3 (8.1%)	
	T2	10 (16.1%)	4 (16%)	6 (16.2%)	
	T3	23 (37.1%)	14 (56%)	9 (24.3%)	
	T4	25 (40.3%)	6 (24%)	19 (51.4%)	
**N (TNM) N (%) **					NS
	N0	17 (27.4%)	8 (32%)	9 (24.3%)	
	N1	7 (11.3%)	2 (8%)	5 (13.5%)	
	N2a	3 (4.8%)	2 (8%)	1 (2.7%)	
	N2b	9 (14.5%)	5 (20%)	4 (10.8%)	
	N2c	19 (30.6%)	6 (24%)	13 (35.1%)	
	N3	7 (11.3%)	2 (8%)	5 (13.5%)	
**Stage**					NS
	I	1 (1.6%)	1 (4%)	0 (0%)	
	II	3 (4.8%)	0 (0%)	3 (8.1%)	
	III	18 (29%)	8 (32%)	10 (27%)	
	IV	40 (64.5%)	16 (64%)	24 (64.9%)	
**Tumor Site N (%) **					NS
	Oral cavity	5 (8.1%)	3 (12%)	2 (5.4%)	
	Oropharynx	42 (67.7%)	17 (68%)	25 (67.6%)	
	Hypopharynx	4 (6.5%)	0 (0%)	4 (10.8%)	
	Larynx	11 (17.7%)	5 (20%)	6 (16.2%)	
**Metastatic Relapse N (%) **					0.025
	No	49 (80.3%)	24 (96%)	25 (69.4%)	
	Yes	12 (19.7%)	1 (4%)	11 (30.6%)	
	NA	1	0	1	
**HPV N (%) **					0.002
	HPV-	27 (43.5%)	5 (20%)	22 (59.5%)	
	HPV+	35 (56.5%)	20 (80%)	15 (40.5%)	
**Anisokaryosis**					<0.001
	Mild	28 (45.2%)	20 (80%)	8 (21.6%)	
	Moderate	19 (30.6%)	5 (20%)	14 (37.8%)	
	Marked	15 (24.2%)	0 (0%)	15 (40.5%)	
**CENP-A H-score**					0.003
	Median (IQR)	1.6 (1.2–2.7)	1.8 (1.6–2.7)	1.5 (0.7–1.8)	
**Local Disease Control at 2 years**					<0.001
	Yes	38 (61.3%)	24 (96%)	14 (37.8%)	
	No	24 (38.7%)	1 (4%)	23 (62.2%)	

NA: not available; NS: not significant.

**Table 2:** Logistic regression to test predictive value for disease control.

		Univariate Analysis			Multivariate Analysis		
Factors	Category	OR	95% IC	*p * -Value	OR	95% IC	*p * -Value
**T (TNM) **				NS			
	T1/T2	1					
	T3/T4	0.56	[0.1; 1.9]				
**N (TNM) **				NS			
	N0	1					
	N+	0.57	[0.2; 1.8]				
**Stage **							
	I/II/III	1		NS			
	IVa or IVb	0.46	[0.1; 1.4]				
**HPV status **				0.02			
	HPV-	1					
	HPV+	3.61	[1.3; 11.0]				
**Anisokaryosis **							
	Mild	1		<0.001	1		0.03
	Moderate	0.29	[0.06; 1.1]		0.74	[0.14; 3.9]	
	Marked	0.03	[0; 0.1]		0.11	[0.01; 0.7]	
**CENP-A H-score **							
		3.1	[1.5; 7.2]	0.005			
**CENP-A pattern **				0.001			0.002
	non-C	1			1		
	C	39.4	[7.1; 744.0]		17.6	[2.6; 362.8]	

Factors with a *p-*value less than 10% in the univariate analysis were included in the multivariate analysis. NS: not significant.

## Data Availability

Data analyzed during this study are included in this manuscript and associated Supplementary Materials.

## Supplementary Materials

The following are available online at https://www.mdpi.com/article/10.3390/cancers13163928/s1. Supplementary Methods describing, as stated in the main manuscript file, the methods for detailed processing of tissue sections for immunohistochemistry staining; immunofluorescence staining and image acquisition and analysis; xenografts and irradiation; Supplementary References from Supplementary Methods; Supplementary Figures S1–S6 with their corresponding legends displayed below each figure; Supplementary Tables S1 and S2 with their legends displayed below each table.
