# Supplementary material for: CENP-A Subnuclear Localization Pattern as Marker Predicting Curability by Chemoradiation Therapy for Locally Advanced Head and Neck Cancer Patients
Source: Cancers (Basel). 2021 Aug 4;13(16):3928. doi: 10.3390/cancers13163928 (PMC8391827; doi:10.3390/cancers13163928)
Supplement: Supplementary file 1 [file cancers-13-03928-s001.zip › cancers-1276708 -SUPPLEMENTARY -done_revised.pdf]

cancers MDPI

Supplementary materials

CENP-A subnuclear localization pattern as marker predicting curability by chemoradiation therapy for locally advanced head and neck cancer patients

Pierre Verrelle, Didier Meseure, Frédérique Berger, Audrey Forest, Renaud Leclère, André Nicolas, Emilie Fortas, Xavier Sastre-Garau, Marick Lae, Sabah Boudjemaa, Rodrigue Mbagui, Valentin Calugaru, Dalila Labiod, Leanne De Koning, Geneviève Almouzni and Jean-Pierre Quivy

Supplementary informations are provided as a single .pdf file with the following contents:

- Supplementary Methods describing as stated in the main manuscript file the methods for detailed processing of tissue section for immunohistochemistry staining; immunofluorescence staining and image acquisition and analysis; Xenografts and irradiation
- Supplementary References from Supplementary methods
- Supplementary figures S1 to S6 with their corresponding legend displayed below each figure
- Supplementary Table S1 and Supplementary Table S2 with their legends displayed below

Supplementary Methods

Immunohistochemistry staining

Tissue sections were deparaffinized and rehydrated through a series of xylene and ethanol washes. Briefly, key steps included: (i) antigen retrieval in 0.1 M citrate buffer, pH=6 (Biocare) in a pressure cooker (4 minutes); (ii) blocking of endogenous peroxidase activity by immersing sections in 3% hydrogen peroxide in methanol for 15 minutes and subsequently rinsing them in water and PBS; (iii) incubation with primary antibodies against the targeted antigen; (iv) immunodetection with a biotin-conjugated secondary antibody formulation that recognizes rabbit and mouse immunoglobulins, followed by peroxidase-labeled streptavidin and linking with a rabbit biotinylated antibody against mouse immunoglobulin G (DAKO SA), and (v) chromogenic revelation with DAB and counterstaining with Mayer's hematoxylin.

immunofluorescence staining

For immunofluorescence analysis of breast samples and xenografts, we collected tissue and incubated them for 1 hour at 4°C in PBS complemented with 30% sucrose for cryopreservation, embedded in tissue freezing medium (Leica) and frozen in isopentane cooled by liquid nitrogen. We used 20 um cryosections using a cryostat (Leica) on superfrost plus slides and immediately fixed with 2% paraformaldehyde in PBS for 20 minutes at room temperature. Following fixation, the tissue cryosections were incubated for 45 min in PBS supplemented with 5% fœtal bovine serum and 0.3% TritonX-100 followed by incubation with the anti-CENP-A used for immunohistostaining (Cell Signaling #2186, Rabbit, Polyclonal, 1/1000) in PBS 1% BSA 0.3% TritonX-100 overnight 4°C. Following 3x10 min washes in PBS supplemented with 0.1% Tween20 sections were incubated for 1 hour at RT with the Alexa fluor conjugated secondary antibody. After 3x10 min washes in PBS 0,1% Tween20, nuclei were stained by DAPI, washed 3x10 min in PBS and mounted in Vectashield on microscopic slide. Immunofluorescence images were acquired using an

LSM780 confocal microscope and a Zeiss Imager Z1 epifluorescence microscope piloted with Metamorph software, a x63 oil objective lens and an ORCA-Flash4.0 LT PLUS Digital CMOS camera (Hamamatsu). We used Fiji software for Z projection with maximal intensity of Z-stacks (0.2 micron). We quantified the number of CENP-A foci, with the 3D-FIED macro [1] from Z-stacks images acquired with the Z1 epifluorescence microscope.

Xenografts and irradiation

SCC61 and radioresistant SQ20B cell lines were derived from human HNSCC [2], and kindly provided by Dr V. Favaudon (Institut Curie, Orsay). Eight to nine-weeks-old female nude NMRI mice (Janvier labs, Le Genest-Saint-Isle, France) were used throughout the study in line with the ARRIVE guidelines. Mice were housed in a controlled enriched environment with food and water available ad libitum. An acclimatization period of one week was respected between mice arrival and the start of the experimental protocol to allow mice to get used to their new environment and thus reduce their stress. Xenografted tumors were obtained by subcutaneous injection of $4x10^6$ SCC61 or SQ20B cells suspended in 40 µL of PBS in the mouse right thigh**.** Tumors were measured with a digital caliper, and tumor volumes were calculated using the following formula: length × width × width/2. Experimental irradiations were performed using a XRAD 320 biological X rays (20 MA, 200 KV) irradiator (Precision X-Ray, Accela, North Branford, CT, USA). A fractionated 20 Gy tumor irradiation was delivered for five consecutive days (5 X 4 Gy) when a tumor volume of 250 to 400 mm3 was evidenced. Mice were integrated into the experimental groups so that the average tumor volume was equivalent between the irradiated group and the control group. Mice were identified by a permanent marking method to avoid confounders. Mice were weighed and tumor volumes were measured twice a three times a week. For ethical reasons, the animals were sacrificed when tumors reached 2000 mm3 (humane endpoint). Experimental procedures were specifically approved by the ethics committee of the Institut Curie CEEA-IC #118 (Authorization #21973-2019091218441437 v1- given by National Authority) in compliance with the international guidelines.

Supplementary References

1 Cantaloube, S., Romeo, K., Le Baccon, P., Almouzni, G. & Quivy, J. P. Characterization of chromatin domains by 3D fluorescence microscopy: An automated methodology for quantitative analysis and nuclei screening. *BioEssays* **34**, 509–517 (2012).

2 Weichselbaum, R. R. et al. Radiation-resistant and repair-proficient human tumor cells may be associated with radiotherapy failure in head- and neck-cancer patients. Proc Natl Acad Sci U S A 83, 2684–2688 (1986).

Supplementary Figures

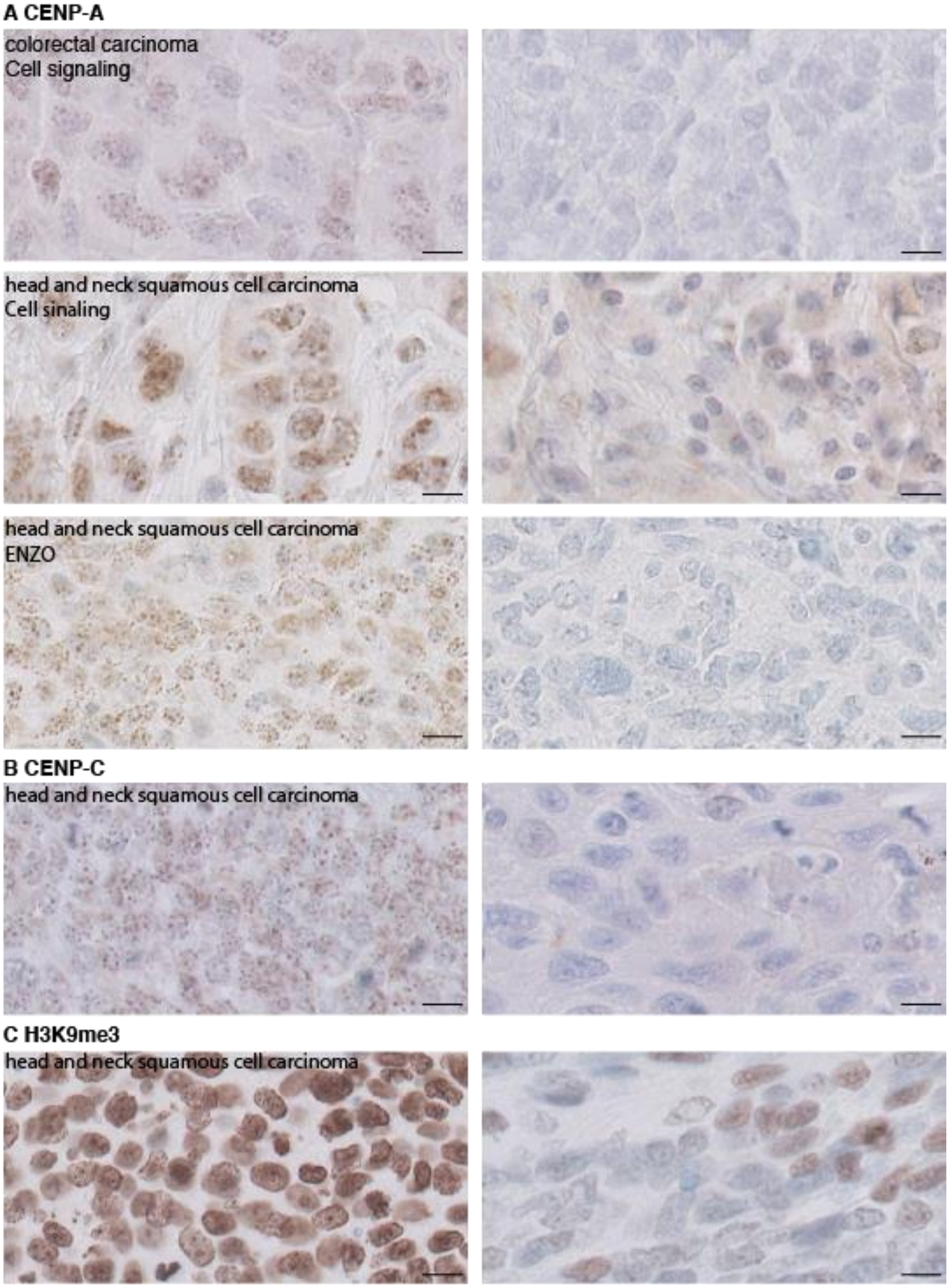

Figure S1. AFA but not formol fixation reveals CENP-A by Immunohistochemistry staining. Immunohistochemical staining of carcinomas fixed with AFA (left) or formol (right). **A**) Detection of CENP-A in colorectal carcinoma and head and neck squamous cell carcinoma with two distinct antibodies as indicated (Cell signaling and ENZO). **B**) Detection of CENP-C in head and neck squamous cell carcinoma. **C**) as B) but H3K9me3. Scale bar is 10 µm.

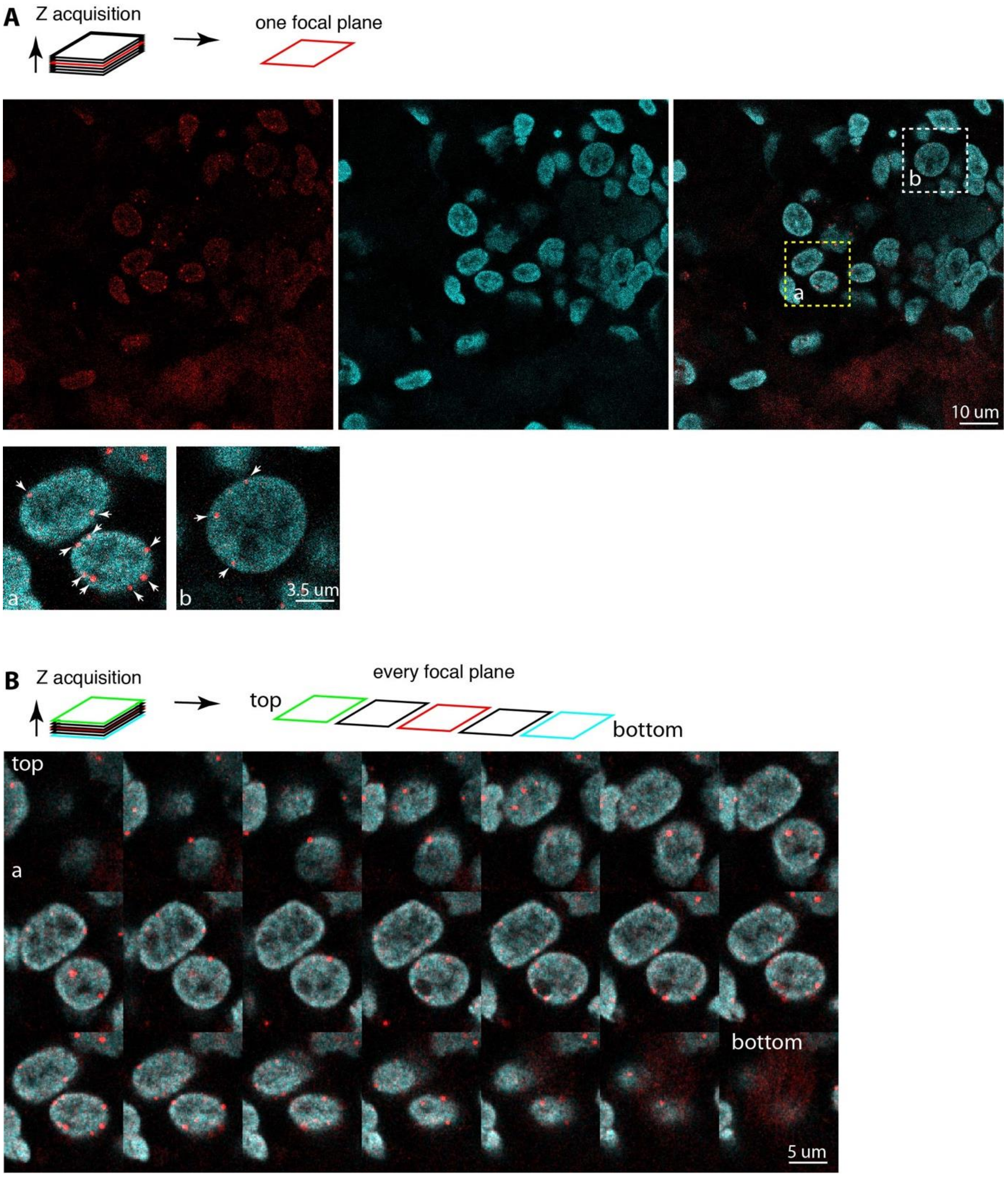

Figure S2. CENP-A staining by immunofluorescence in normal human breast tissue. A) Top: Confocal image of CENP-A staining (red) on normal breast tissue cryosection. DAPI (blue) and merge images are shown. Bottom insets show 3x magnifications of the merge image of the a and b areas boxed in dashed line above. Arrow heads point to CENP-A foci. Scale bars are indicated. **B**) Consecutive confocal sections from the acquisition in z of the nuclei corresponding to the area a from above. Scale bar is indicated.

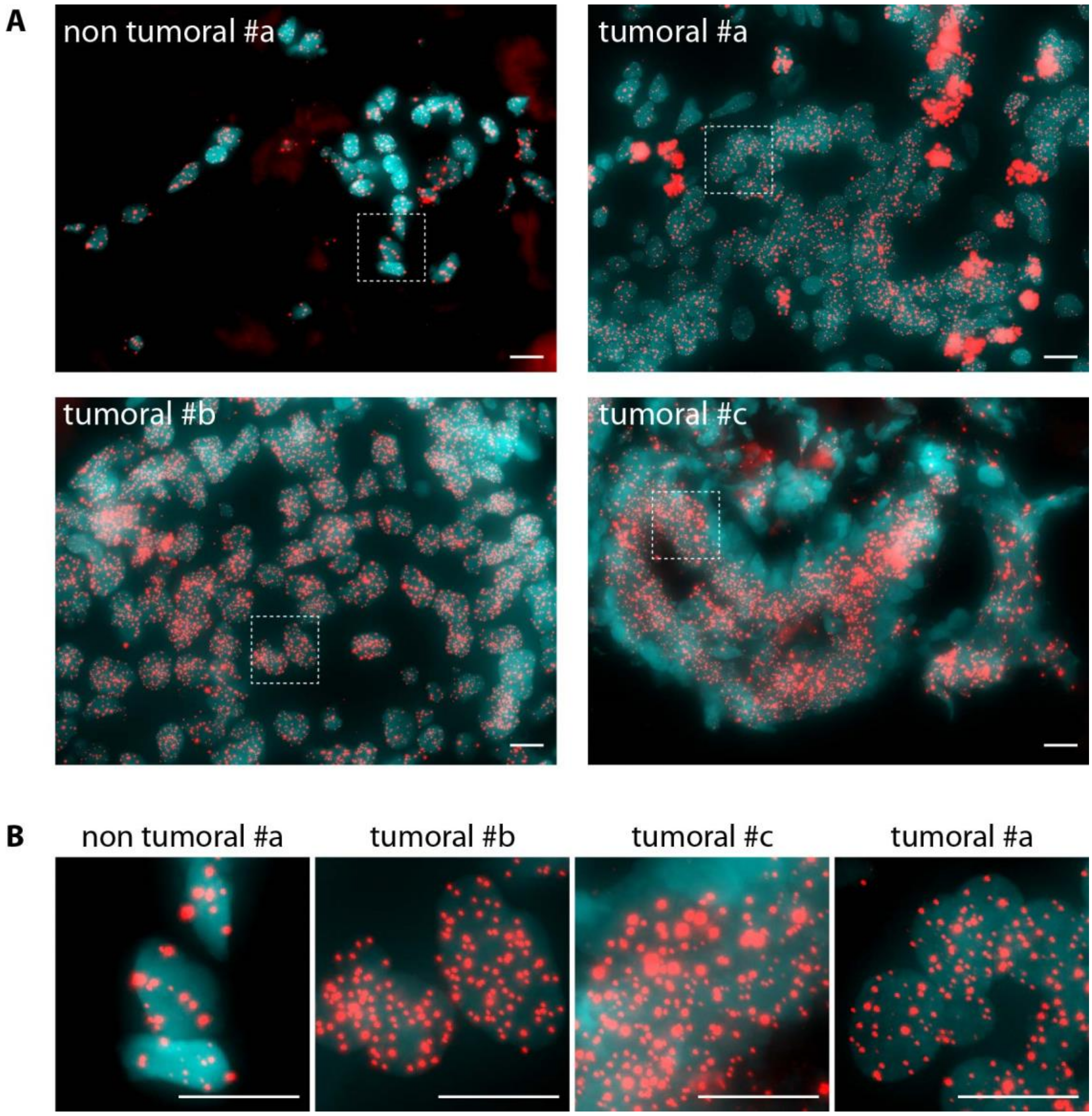

Figure S3. CENP-A nuclear localization in normal and tumoral breast revealed by immunofluorescence in cryosections
A) Top: images of z projection with maximum intensity from 3D acquisition of immunofluorescence CENP-A staining (Red) in human breast tissue. Nuclei are Stained with DAPI (Blue) and scale bar is 10 µm. Non-tumoral and tumoral tissue sample from the same patient (#a) and two other tumors from distinct patients are shown (#b and #c) as indicated. **B**) Bottom: insets showing magnification of the areas boxed in dashed line above. Scale bar is 10 µm.

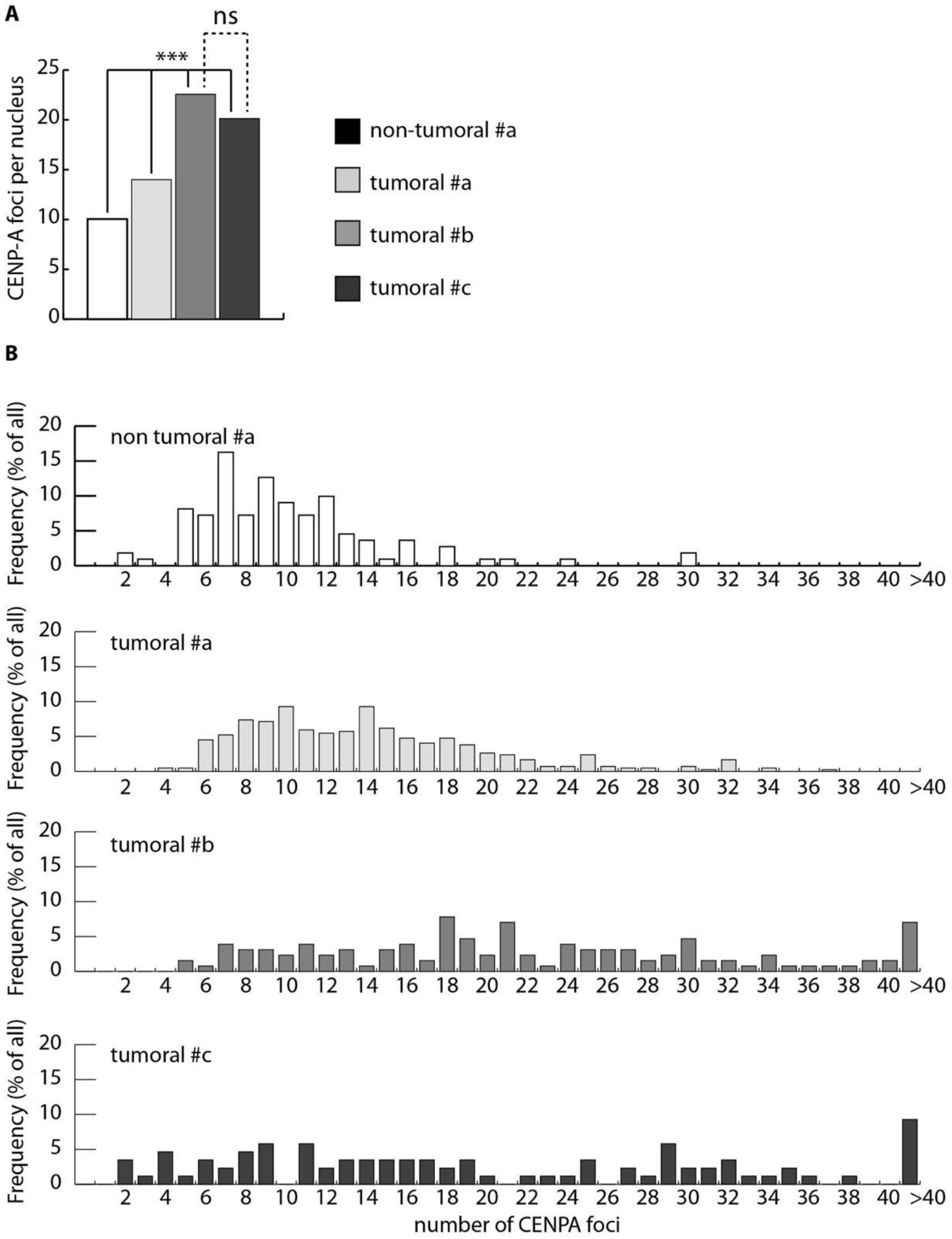

Figure S4. the number of CENP-A foci increase in breast tumors. A) Bar plot graph showing the mean number of CENP-A foci per nucleus quantified from Z acquisition from non-tumoral tissue sample #a (n= 111 nuclei) and tumoral tissue samples #a (n= 420 nuclei), #b (n= 128 nuclei) and #c (n= 86 nuclei) from figure S3. ***: $p<0.001$; ns: non-significant (Mann-Whitney). **B**) Bar plot graph showing the distribution of the number of CENP-A foci per nuclei in samples #a, #b and #c from above.

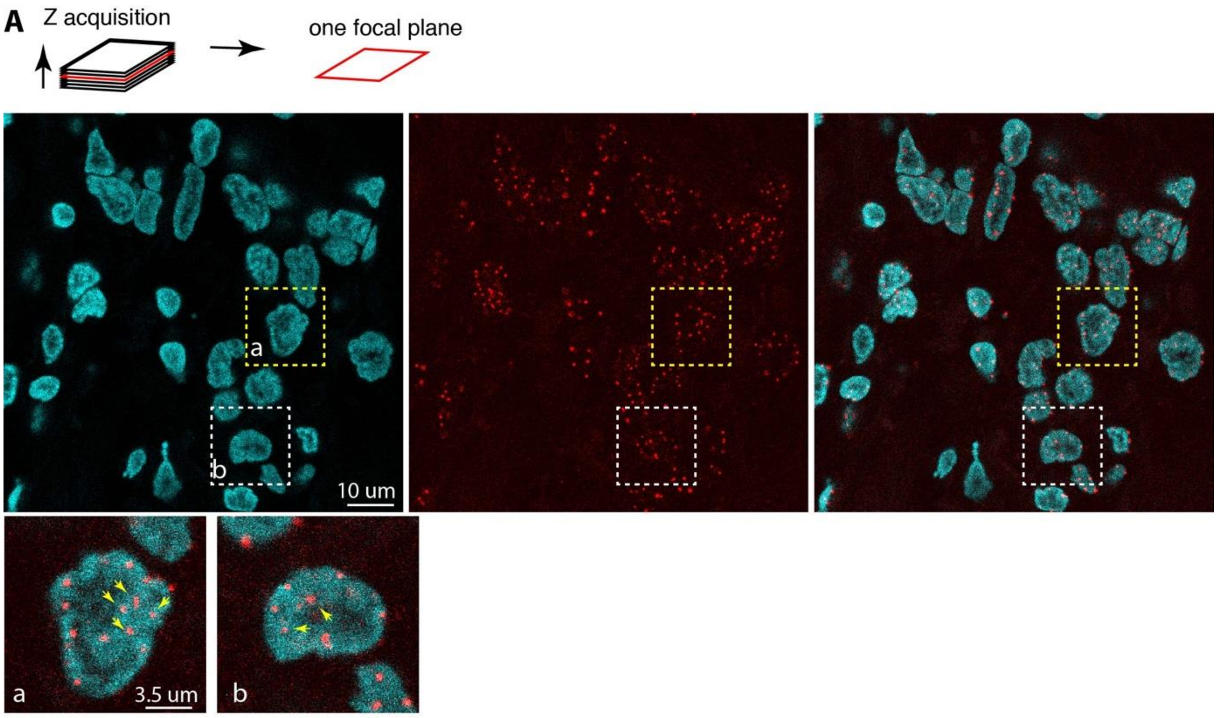

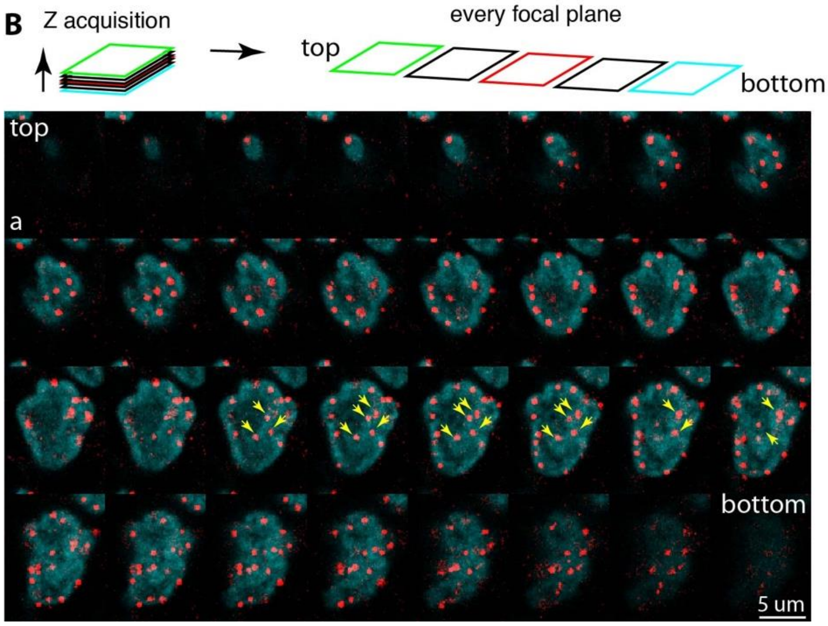

Figure S5. CENP-A staining by immunofluorescence staining in breast tumor. A) Confocal image of CENP-A staining (red) on tumoral breast tissue cryosection corresponding to patient #a in supplementary figure 1 and figure 3. DAPI (blue) and merge images are shown. Bottom insets show 3x magnifications of the merge image of the a and b areas boxed in dashed line above. Yellow arrowheads point to CENP-A foci. Scale bars are indicated. **B**) Consecutive confocal sections from the nuclei in area boxed in yellow (nuclei a). Yellow arrowheads point to CENP A foci that are not localized at the nuclear periphery but inside the nucleus.

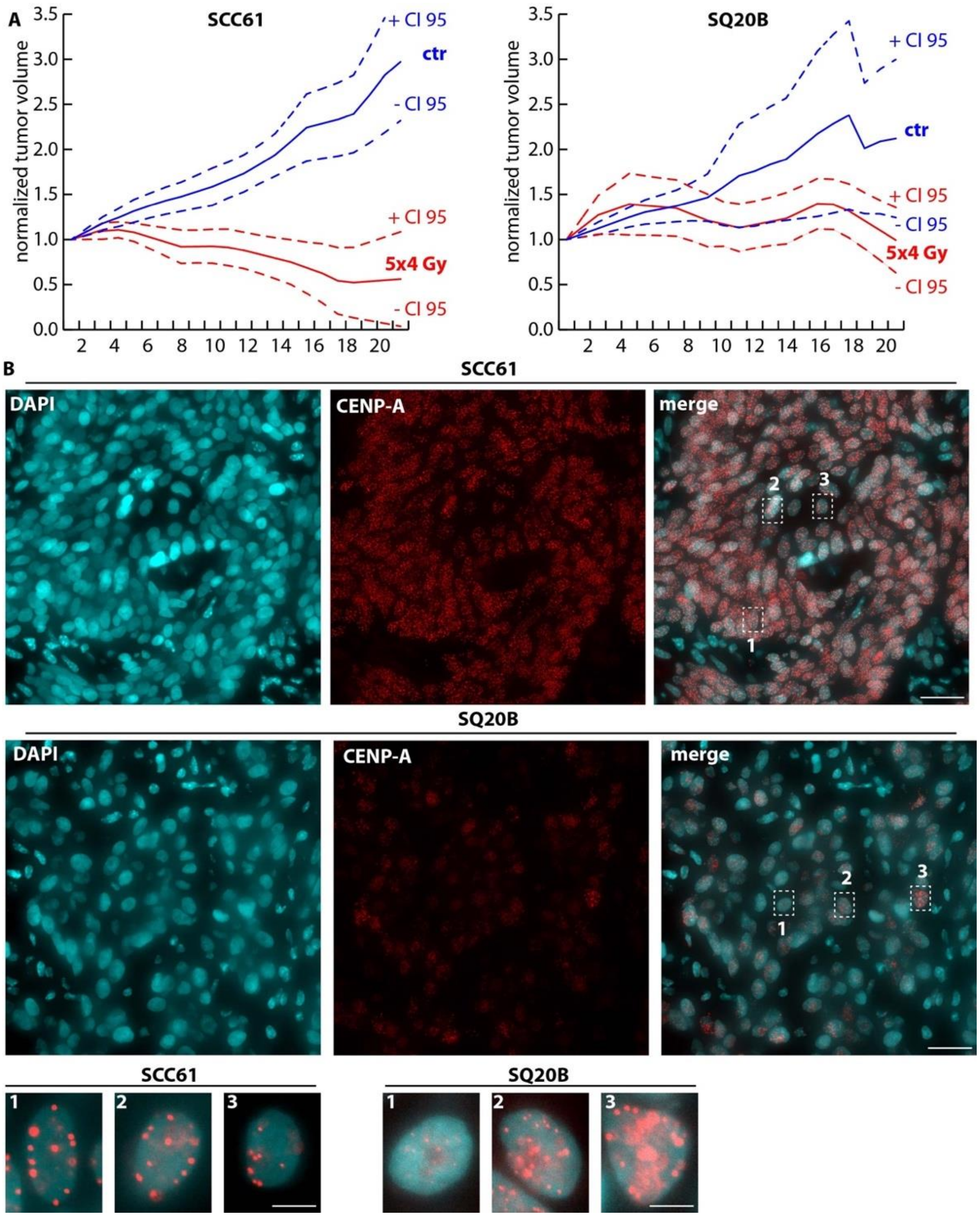

Figure S6. CENP-A localization in SCC61 and SQ20B grafted cells. A) Curve diagram showing normalized average tumor volume from grafted cells as a function of time for the radio sensitive cell line SCC61 (control, n=8 mice; 5x4 Gy n=8 mice) and the radio resistant cell line SQ20B (control: n=8 mice; 5x4 Gy n=8 mice). Blue, control (ctr) cells, Red (5x4 Gy) following irradiation. N=8 mice for each conditions (sample size was defined using a logrank test on InVivoStat statistical software). The dashed lines represent the 95% confidence interval. For each cell line, the curves were compared using a t-test (Prism-GraphPad) and are statistically significantly different for SCC61 cells (p=0.0001). **B**) Top. images of z projection with maximum intensity from 3D acquisition of immunofluorescence CENP-A staining (Red) in cell lines as indicated on top. DAPI and merge images are shown. Scale bar is 50 μm. Bottom. Insets show magnification of the dashed area in the merge image. Scale bar is10 μm.

Supplementary Tables

Table S1. correlations between disease control and patients and tumor characteristics. NS: non significant.

Characteristics		Total (N=62)	Local disease control at 2 years Yes (N = 38)	No (N =2 4)	Test
Age (yr)					NS
	Median (IQR)	62.(56–68)	61 (56–66)	65 (56–70)	
Ki67 (%)					NS
	Median (IQR)	60 (35–75)	60 (35–75)	67.5 (37.5–67.5)	
Gender - N (%)					NS
	F	10 (16.1%)	7 (18.4%)	3 (12.5%)	
	M	52 (83.9%)	31 (81.6%)	21 (87.5%)	
T (TNM) - N (%)					NS
	T1	4 (6.5%)	3 (7.9%)	1 (4.2%)	
	T2	10 (16.1%)	7 (18.4%)	3 (12.5%)	
	T3	23 (37.1%)	17 (44.7%)	6 (25%)	
	T4	25 (40.3%)	11 (28.9%)	14 (58.3%)	
N (TNM) - N (%)					NS
	N0	17 (27.4%)	12 (31.6%)	5 (20.8%)	
	N1	7 (11.3%)	5 (13.2%)	2 (8.3%)	
	N2a	3 (4.8%)	2 (5.3%)	1 (4.2%)	
	N2b	9 (14.5%)	6 (15.8%)	3 (12.5%)	
	N2c	19 (30.6%)	9 (23.7%)	10 (41.7%)	
	N3	7 (11.3%)	4 (10.5%)	3 (12.5%)	
Stage					NS
	I	1 (1.6%)	1 (2.6%)	0 (0%)	
	II	3 (4.8%)	1 (2.6%)	2 (8.3%)	
	III	18 (29%)	14 (36.8%)	4 (16.7%)	
	IVa or IVb	40 (64.5%)	22 (57.9%)	18 (75%)	
tumor site - N (%)					NS
	Oral cavity	5 (8.1%)	4 (10.5%)	1 (4.2%)	
	Oropharynx	42 (67.7%)	24 (63.2%)	18 (75%)	
	Hypopharynx	4 (6.5%)	1 (2.6%)	3 (12.5%)	
	Larynx	11 (17.7%)	9 (23.7%)	2 (8.3%)	
Metastatic relapse - N (%)					<0.001
	No	49 (80.3%)	37 (97.4%)	12 (52.2%)	
	Yes	12 (19.7%)	1 (2.6%)	11 (47.8%)	
	NA	1	0	1	
Induction therapy - N (%)					NS
	No	38 (61.3%)	26 (68.4%)	12 (50%)	
	Yes	24 (38.7%)	12 (31.6%)	12 (50%)	
RT +/- CT - N (%)					NS
	RCT	42 (67.7%)	27 (71.1%)	15 (62.5%)	
	RT	20 (32.3%)	11 (28.9%)	9 (37.5%)	
RT stop >5j - N (%)				>5j	NS
	No	55 (88.7%)	33 (86.8%)	22 (91.7%)	
	Yes	7 (11.3%)	5 (13.2%)	2 (8.3%)	
Total dose of RT - N (%)			RT	de	NS
	70 Gy	60 (96.8%)	37 (97.4%)	23 (95.8%)	
	>70 Gy	2 (3.2%)	1 (2.6%)	1 (4.2%)	

HPV - N (%)					0,02
	HPV-	27 (43.5%)	12 (31.6%)	15 (62.5%)	
	HPV+	35 (56.5%)	26 (68.4%)	9 (37.5%)	
Anisocaryosis					<0.001
	Mild	28 (45.2%)	24 (63.2%)	4 (16.7%)	
	Moderate	19 (30.6%)	12 (31.6%)	7 (29.2%)	
	Marked	15 (24.2%)	2 (5.3%)	13 (54.2%)	
CENP-A H-score					<0.001
	Median (IQR)	1.6 (1.2–2.7)	1.8 (1.6–2.7)	1.2 (0.6–1.6)	
CENP-A pattern					<0.001
	C	25 (40.3%)	24 (63.2%)	1 (4.2%)	
	non-C	37 (59.7%)	14 (36.8%)	23 (95.8%)	

Table S2. T(TNM), N(TNM) and stage of the tumors at the specific localization sites.

Characteristics	Class	Oral cavity N(%)	Oropharynx N(%)	Hypopharynx N(%)	Larynx N(%)
T (TNM)					
	T1	0 (0%)	4 (9.5%)	0 (0%)	0 (0%)
	T2	2 (40%)	7 (16.7%)	0 (0%)	1 (9.1%)
	T3	2 (40%)	11 (26.2%)	2 (50%)	8 (72.7%)
	T4	1 (20%)	20 (47.6%)	2 (50%)	2 (18.2%)
N (TNM)					
	N0	1 (20%)	7 (16.7%)	1 (25%)	8 (72.7%)
	N1	1 (20%)	4 (9.5%)	1 (25%)	1 (9.1%)
	N2a	0 (0%)	2 (4.8%)	1 (25%)	0 (0%)
	N2b	0 (0%)	9 (21.4%)	0 (0%)	0 (0%)
	N2c	2 (40%)	15 (35.7%)	1 (25%)	1 (9.1%)
	N3	1 (20%)	5 (11.9%)	0 (0%)	1 (9.1%)
Stage					
	I	0 (0%)	1 (2.4%)	0 (0%)	0 (0%)
	II	0 (0%)	2 (4.8%)	0 (0%)	1 (9.1%)
	III	2 (40%)	7 (16.7%)	2 (50%)	7 (63.6%)
	Iva or Ivb	3 (60%)	32 (76.2%)	2 (50%)	3 (27.3%)
